# Adherence to treatment in patients with delusional disorder - study of acute inpatient population in psychiatry ward between 2007-2017

**DOI:** 10.1192/j.eurpsy.2021.1444

**Published:** 2021-08-13

**Authors:** A. Delgado, D. Barbosa, R. Curral

**Affiliations:** 1 Psychiatry, Centro Hospitalar Universitário de São João, Porto, Portugal; 2 Psychiatry, Sao Joao Hospital and University Centre, Porto, Portugal

**Keywords:** Delusional disorder, treatment adhesion

## Abstract

**Introduction:**

Delusional disorder is a mental illness in which delusions are the dominant symptom. Delusional disorder is not well studied relative to other psychotic disorders - it is poorly understood in practically every aspect of its nature, including cause, phenomenology, prevalence, comorbidity, course, treatment, and prognosis.

**Objectives:**

To study the clinical and sociodemographic characteristics of individuals admitted for inpatient treatment with the diagnosis of delusional disorder, in particular the adherence to treatment.

**Methods:**

Retrospective observational study of inpatient treatment of patients with delusional disorder diagnosis between january 1^st^2007 and 31^th^ december of 2017 in the Psychiatry Service of CHUSJ. Follow up of 2 years from discharge. Data collected included sociodemographic characteristics and clinical features. Descriptive analysis of the results was performed using SPSS (v.26).

**Results:**

In the period of time analyzed, 152 hospitalizations were identified, corresponding to 114 patients: 38.2% male and 62.8% female. The average age was 58 years. 3 months after discharge: 65% of patients were going to the medical appointments, which dropped to 60% in 6 months, 55% in 12 months, 53% in 12 and 24 months. Regarding adherence to the treatment: 65% of patients were still adherent to medication in 3 months time, 55% in 6 months, dropping to 50% in a year and to 48% in 2 years. There is a relation between involuntary discharge and adherence to consultations and medication.

**Conclusions:**

A cardinal characteristic of delusional disorder, conviction that one is not mentally ill, contributes complexity to the treatment challenges and profoundly affects the therapeutic relationship.

